# Professionalism in practice: Exploring the ethical perplexity of involving students in Medical Education Research

**DOI:** 10.30476/jamp.2020.74921.0

**Published:** 2020-10

**Authors:** DINESH KUMAR. V, MAGI MURUGAN

**Affiliations:** 1 Department of Anatomy, Jawaharlal Institute of Postgraduate Medical Education and Research, Puducherry, India; 2 Department of Anatomy, Pondicherry Institute of Medical Sciences, Puducherry, India

**Keywords:** Ethics, Professionalism, Medical education, Research, Recruitment

## Abstract

With the worldwide increase in the number of medical education researches, few ethical quagmires have emerged regarding the recruitment and inclusion of students in research projects. The ethicists often tend to raise questions regarding the possibilities of students getting coerced to participate, such as whether they receive extra course credits in exchange for their participation, or whether their privacy is getting violated in the course of data collection. It is the need of the hour to address the perplexity behind these ethical dilemmas. Some answers to the ethical questions might call for implication of change in the organization of research, thereby affecting the output. This commentary tries to address these issues in a genuine manner and affords a way forward in the context of ethics related to educational research. By treading the delicate path between framing the research question which never encompass any ethical breaches and compromising the rigour of the study design to suffice certain baseless hindrances, we could appreciate the importance of practical ethics in educational researches

## Introduction

One of the core tenets of obtaining informed consent in researches involving human subjects is that the potential research subjects should not feel in any way coerced to participate. The participants should always possess the due rights of withdrawing from the research pursuit without any compromise on the service being provided to them. The onus of maintaining the ethical integrity in medical education researches involving students is even more because the researcher who is a faculty member is often involved in imparting his or her learning and evaluation ( 1 ). On one hand, students constitute a fertile field for conducting researches related to pedagogy, curriculum evaluation and qualitative researches based on reflections. On the other hand, their autonomy in participation or competence to refuse might be potentially restricted ( 2 ). In practice, the ambiguity gets wider when the faculty member conducts an educational evaluation solely for institutional program development and as a result gets a sizeable piece of generalized knowledge which is appropriate for a scholarly publication ( 3 ). 

**Ethical Quagmire of educational researches**

Institute review board should be clear in defining the blurring boundary between scholarly education practices which are innovative, self-observing and aim at advancing the students’ learning process ( 4 ) and differentiating it from medical education research with the main aim of generating an evidence for advancing the wider knowledge of the discipline, as such. For example, let us consider faculty member A, who is interested in delivering his lecture in a modified way and at the end of the lecture obtains an anonymous feedback from the students using audience response system. This can be considered under the purview of scholarly teaching practice, as the primary intent of the teacher is advancement of the students. Another faculty member B, randomly divides the students into two groups and runs the class using the same modified way, maintaining the rest of students as control. Eventually, he conducts an assessment to evaluate the effectiveness of the modified teaching methodology. This should be considered as a medical education research because the primary intention of the teacher over his or her is evaluating the teaching methodology, which would contribute to the advancement of the generalized knowledge of the discipline, compromising the learning process of a subpopulation of the students. Nevertheless, the points at which internally directed evaluations become publicly shared scholarships is often murky, and it varies according to the philosophy of the disciplines and institutional policies. 

Secondly, in time-consuming research assessments such as a longitudinal follow-up study design, the course of the study might distract the students from their ulterior focus on education ( 5 ), thereby posing risks which are not evident for the participants, researchers and sometimes the review boards as well. Thirdly, in close knit communities such as medical schools, it is not uncommon for a student, who readily expresses his/her willingness to participate in educational research, to expect privileges in examinations and opportunities ( 2 ). This factor, per se, cannot be monitored by the review boards and it is the ethical obligation of the researcher to provide equitable opportunities to all students and avoid possible biases between the participants and others. Fourthly, when sensitive issues pertaining to student participants such as debt burden, sexual inclination, psycho-social predilection, etc. are collected by the faculty member who beholds the powerful authority, the potential risks of researches get compounded ( 6 ) and the authentic voluntarism get compromised. Despite all possibilities, a study by *Forester* et al. ( 7 ) showed that 93% of students felt that medical education researches are essential for improving their own education, and 91% reported that they hadn’t felt any palpable coercion arising out of faculty authority. 

**Resolving the professional perplexity:**

It can be argued that educational researches have to gear to the idiosyncrasies of the educational domains and unlike other conventional researches, controlled experimentation need not always be the preferred method ( 8 ). In addition, the reciprocation of students towards educational experiments differs across the world. Especially, when students tend to be passive and uncritical learners, such as Asian classroom environments, the teachers possess unquestionable authority over students by virtue of being sole knowledge providers ( 9 ). In such conditions, students need to be informed regarding their “autonomy” to answer and the need for involving students should be justified based on ethical principles namely, beneficence and justice. The study design should be weighed based on risk -benefit ratio and if it involves convenient selection of students, equitable selection should be ensured as some students may perceive themselves disadvantaged if unable to participate ( 10 ). Some researchers do offer a course credit as an alternate to cash coupons for participation in researches and this practice constitutes another ethical quandary for review boards ( 11 ). This practice, even though not amounting to coercion threat, has possibilities of unbalancing the existing fairness of the system by providing undue advantage to the participants.

In some endeavours, where the researcher had to correlate the retrospective data available in the institute, students might not have been aware regarding the sensitivity attached to the dataset at that particular point of time. For example, a student giving an informed consent for a qualitative study involving data related to loan indebtedness or parental occupation might feel stigmatized in the due course. In such case, blinding the data by removing the specific identifiers, maintaining confidentiality, and using pseudonyms become necessary to entitle the protection of the students’ rights. It becomes complex from ethical perspective when a researcher, out of moral obligation, intends to help a student based on the sensitive data he/ she had provided in the course of research by compromising the confidentiality ( 12 ). A more valid solution is deploying student representatives and considering their views on Institutional Review Boards (IRBs) which would bring the stakeholder perspective related to the sensitivity of a particular topic ( 2 ). 

A delicate area related to this is the subconscious surfacing of the role conflicts when a faculty conducts a research ( 3 ). Consider an example, where a program director who wishes to analyze the elements of an educational context which would potentially foster or pose barriers to learning. For this, he assigns the students randomly into two groups and in the process he needs to place some students in the context where they might not learn the best. This eventually places him in an ethical bind because, as a researcher, he might not be able to respond well to the ulterior needs of those students in order to successfully accomplish the study, and as a program director, he needs to place the academic well-being of the students ahead. In the second example, when a study pursued by a teacher-researcher suffers from high attrition, he would sense an ethical dilemma between adhering to authentic volunteerism and compromising the validity of the study. 

**Achieving fairness in educational research: Lessons from our experience**

The role of IRBs in educational researches is delicate because sometimes they have to evaluate the research proposals that are “beyond their scope” and ascertain whether the research poses minimal or no risk ( 13 ). At the same time, quality of research should not be made to compromise due to ostensible over-regulations and it should not get influenced by institutional concerns factors as liability. Coercion can only be defined in perceptive grounds of the participants and its construct should be considered under two separate areas: perception of freedom of choice and perception of acceptability of choices ( 14 ). These can be ensured when students know their specified role and rights while participating in educational research. This holds sense in academic environments, where the research investigators and examiners are the same and students might compromise their rights for the sake of appeasing them. In such cases, blinding the views/perspectives of the students and collecting it in an anonymised manner would help to serve the purpose. IRBs should analyze the medical education research proposal under two subdivisions ( 15 ): *procedural justice*, whereby the proposal is scrutinized for consistency, lack of bias and adherence to basic ethical standards, and *interactional justice*, whereby the behavioural aspects of decision process are ascertained and for this it would be optimal to include a broader range of stakeholders including research participants ( 16 ). In other words, attempts should be made to weigh the potential balance between interpersonal sensitivity involved upon conducting the research and justification given by the researcher. 

Two more intricacies occur when medical educators set out to conduct classroom evaluations with the goal of improving educational programs, but later they realize that the information they have gathered constitute a generalizable knowledge beneficial for peers and share them with others via publication or conference ( 17 ). For example, an educator is entitled to obtain feedback from the students regarding his/her module and this information might sometime reveal a new perspective/trend. If the educator feels that this piece of information could be disseminated to wider audience, it could be retrospectively thought of and weighed based on similar publications which have been published previously. This props up two issues: 1) Does disseminating this piece of information warrants consent from the providers i.e. students? 2) Why can't it be taken as an easy go passage by some, if not all. Firstly, it is difficult to determine the intention of those who are engaged in evaluations, and second the data generated by evaluation programs need not be generalizable in greater degrees in other institutes, lacking the criteria for scientific knowledge of that discipline. Some IRBs consider these curricular evaluations for retroactive approval on the basis that faculty members who are interested in scholarships of teaching have legitimate interest in students, though lacking the rigorous research skills and these evaluations neither deviate much from standard practice nor involve any recruitment practices. 

## Conclusion

Attempt to improve the learning outcomes of students is fundamental to medical education. However, this process often poses distinct ethical quandaries that are seldom addressed because of the difficulties in determining the intention behind the attempts. We believe that conducting educational evaluations is often a means to begin the process of research socialization and hope that this article will reveal a documentation regarding ethical dilemmas when a faculty member intends to conduct medical education researches. Irrespective of the issues mentioned above, any faculty member should be encouraged to conduct high quality evaluations in corresponding disciplines and disseminate the findings to all stakeholders, adding value to the medical education scholarship. This requires a concrete triangulation between institutes, faculty and students, which would further the ethics-based education endeavours worldwide. 

## References

[1] Ferguson LM, Myrick F, Yonge O ( 2006). Ethically involving students in faculty research. Nurse Education Today.

[2] Christakis N (1985). Do medical student research subjects need special protection?. IRB.

[3] Roberts LW, Geppert C, Connor R, Nguyen K, Warner TD (2001). An invitation for medical educators to focus on ethical and policy issues in research and scholarly practice. Acad Med.

[4] Glassick CE, Huber MT, Maeroff GI Scholarship Assessed: Evaluation of the Professorate. San Francisco, CA: Jossey-Bass; 1997.

[5] DuBois JM ( 2002). When is informed consent appropriate in educational research? Regulatory and ethical issues. IRB.

[6] Burgess RG The Ethics of Educational Research. New York: Falmer Press; 1989.

[7] Forester JP, McWhorter DL ( 2005). Medical students’ perceptions of medical education research and their roles as participants. Acad Med.

[8] van der Vleuten CP, Dolmans DH, de Grave WS, van Luijk SJ, Muijtjens A, Scherpbier AJ, et al ( 2004). Education research at the Faculty of Medicine, University of Maastricht: fostering the interrelationship between professional and education practice. Acad Med.

[9] Cortazzi M, Jin LX Culture of learning: language classrooms in China. In: Coleman H, editor. Society and the language classroom. Cambridge: Cambridge University Press; 1996.

[10] Bradbury-Jones C, Alcock J ( 2010). Nursing students as research participants: A framework for ethical practice. Nurse Education Today.

[11] Klitzman R ( 2013). How IRBs view and make decisions about coercion and undue influence. J Med Ethics.

[12] Roberts L, Hardee JT, Franchini G, Stidley CA, Siegler M ( 1996). Medical students as patients: a pilot study of their health care needs, practices, and concerns. Acad Med.

[13] Mueller JH ( 2007). Ignorance is neither bliss nor ethical. North-western University Law Review.

[14] Leak GK ( 1981). Student perception of coercion and value of participation in psychological research. Teaching of Psychology.

[15] Reeser JC, Austin DM, Jaros LM, Mukesh BN, McCarty CA ( 2008). Investigating Perceived Institutional Review Board Quality and Function Using the IRB Researcher Assessment Tool. J Empir Res Hum Res Ethics.

[16] Anderson JA, Sawatzky-Girling B, McDonald M, Pullman D, Saginur R, Sampson HA, et al ( 2011). Research ethics broadly writ: beyond REB review. Health Law Rev.

[17] Mavis BE, Henry RC ( 2005). Being uninformed on informed consent: a pilot survey of medical education faculty. BMC Medical Education.

